# The Molecular Dynamics of *Trypanosoma brucei* UDP-Galactose 4′-Epimerase: A Drug Target for African Sleeping Sickness

**DOI:** 10.1111/j.1747-0285.2012.01392.x

**Published:** 2012-08

**Authors:** Aaron J Friedman, Jacob D Durrant, Levi C T Pierce, Thomas J McCorvie, David J Timson, J Andrew McCammon

**Affiliations:** 1 Biomedical Sciences Graduate Program, University of California San DiegoLa Jolla, CA 92093-0365, USA; 2 Department of Chemistry and Biochemistry, University of California San DiegoLa Jolla, CA 92093-0365, USA; 3 School of Biological Sciences, Queen’s University Belfast, Medical Biology CentreBelfast BT9 7BL, UK; 4 Department of Chemistry and Biochemistry, NSF Center for Theoretical Biological Physics, National Biomedical Computation Resource, University of California San DiegoLa Jolla, CA 92093, USA; 5 Department of Pharmacology, University of California San DiegoLa Jolla, CA 92093, USA; 6 Howard Hughes Medical Institute, University of California San DiegoLa Jolla, CA 92093, USA

**Keywords:** African sleeping sickness, molecular dynamics, protein structure, *Tb*GalE, *Trypanosoma brucei*, UDP-Galactose-4′-Epimerase

## Abstract

During the past century, several epidemics of human African trypanosomiasis, a deadly disease caused by the protist *Trypanosoma brucei*, have afflicted sub-Saharan Africa. Over 10 000 new victims are reported each year, with hundreds of thousands more at risk. As current drug treatments are either highly toxic or ineffective, novel trypanocides are urgently needed. The *T. brucei* galactose synthesis pathway is one potential therapeutic target. Although galactose is essential for *T. brucei* survival, the parasite lacks the transporters required to intake galactose from the environment. UDP-galactose 4′-epimerase (*Tb*GalE) is responsible for the epimerization of UDP-glucose to UDP-galactose and is therefore of great interest to medicinal chemists. Using molecular dynamics simulations, we investigate the atomistic motions of *Tb*GalE in both the *apo* and *holo* states. The sampled conformations and protein dynamics depend not only on the presence of a UDP-sugar ligand, but also on the chirality of the UDP-sugar C4 atom. This dependence provides important insights into *Tb*GalE function and may help guide future computer-aided drug discovery efforts targeting this protein.

Human African trypanosomiasis (HAT), a disease caused by the protist *Trypanosoma brucei* (*T. brucei*), directly affects thousands of sub-Saharan Africans and indirectly places hundreds of thousands more at risk. Current treatments are often ineffective or dangerous, necessitating the development of a new generation of HAT therapeutics.

Disease pathology occurs in two stages. The early hemolymphatic stage is characterized by fever, cephalgia, arthralgia, and pruritus. Current treatments for early-stage HAT include pentamine and suramin. However, pentamine, often used to treat the *T. brucei gambiense* strain, is associated with hypoglycemia and hypotension, and suramin, effective against *T. brucei rhodesiense*, is associated with severe cutaneous reactions, renal failure, anaphylactic shock, and neurotoxicity (1–4).

Once the parasite crosses the blood–brain barrier, the more serious and often irreversible symptoms of the neurological phase are manifest, including disrupted cognition, coordination, and sleep (5). Since 1949, melarsoprol has been the standard treatment for late-stage HAT. Despite its efficacy, a significant number of patients relapse; additionally, 5–10% of those who receive treatment develop severe encephalopathy, often leading to death (6). In the late 20th century, Aventis developed eflornithine, a drug that targets *T. brucei* ornithine decarboxylase. While eflornithine is significantly less dangerous than melarsoprol (7), it is ineffective against the *T. brucei rhodesiense* subspecies (8).

As current therapeutics are problematic, medicinal chemists are actively seeking to identify novel *T. brucei* drug targets. The proteins of the biochemical pathway involved in galactose synthesis are excellent candidates. Although *T. brucei* requires galactose for the synthesis of vital glycoproteins (9), it is unable to intake galactose from the environment. Instead, glucose is acquired via hexose transporters (10) and is subsequently converted to galactose. One of the proteins in the pathway that effectuates this conversion, UDP-galactose 4′-epimerase (*Tb*GalE), inverts the stereochemistry of the UDP-glucose C4 carbon atom to produce UDP-galactose. The two-step reaction proceeds via a transient ketose intermediate and requires rotation of the sugar portion of the ligand for epimerization (Figure 1) (11). Initial drug discovery efforts targeting this protein have identified several promising inhibitors (12). Unexpectedly, virtual screening and subsequent experimental validation identified several agonists as well.

**Figure 1:**
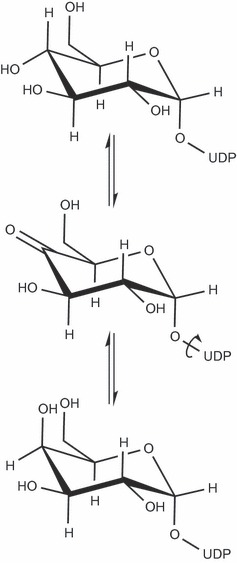
UDP-sugar epimerization. The epimerization reaction proceeds via a UDP-ketose intermediate. Epimerization requires a 180° rotation (‘flip’) of the sugar portion of the ligand.

This study provides insight into the general mechanisms of *Tb*GalE ligand binding. The static lock-and-key model of binding, first proposed by Emil Fischer in 1894 (13), has been largely abandoned in favor of theories that account for receptor flexibility. Specifically, the induced-fit and population-shift models have been much debated over the course of the past 50 years (14). Induced fit suggests that *apo* and *holo* receptors sample distinct regions of conformational space; ligand binding itself induces conformational changes in the receptor (15). Population shift, on the other hand, suggests that an *apo* protein samples many conformations in solution, a subset of which are amenable to ligand binding. Ligands bind to rare but amenable receptor conformations and energetically stabilize them, causing the population of all conformations to shift toward those that can accommodate the ligand (16–19).

Here, we explore the atomistic dynamics of the *Tb*GalE homodimer by investigating the major steps along its reaction coordinate. Using molecular dynamics (MD) simulations, we study the motions of *Tb*GalE homodimer in both the *apo* and *holo* forms, bound to UDP-galactose, UDP-glucose, and the UDP-ketose intermediate. The conformations sampled by the protein depend not only on the presence of a UDP-sugar ligand, but also on the chirality of the UDP-sugar C4 atom. This dependence provides important insight into *Tb*GalE function and may help guide future computer-aided drug discovery efforts targeting this protein.

## Methods and Materials

### System preparation

A crystal structure of *Tb*GalE homodimer [PDB ID: 2CNB (20)] was obtained from the Protein Data Bank (21). To generate missing loops, the structure was aligned to a model of *Tb*GalE that had been created previously (12) based on the 1GY8 *Tb*GalE structure (11). Following alignment, the coordinates of the missing loop atoms were copied from the 1GY8 model. All cocrystallized waters were retained; PDB2PQR (22) and PROPKA (23,24) were used to assign residue protonation states at pH 7.0. Histidine protonation states were visually inspected in VMD (25) to ensure optimal hydrogen bonding. The NAD(H) and Y173 protonation states were manually assigned to properly mimic the active site configuration needed for catalysis (11).

NAD+/NADH parameters were obtained from Walker *et al.* (26,27). Accelerys Discovery Studio 2.5 was used to model the structure of UDP-galactose by changing the fluorine atom of the 2CNB UDP-4-deoxy-4-fluoro-alpha-D-galactose ligand to a hydroxyl group. UDP-glucose and the UDP-ketose intermediate were built by altering the stereochemistry and hybridization of the UDP-galactose C4 carbon atom. Hydrogen atoms were added to the three UDP-sugars using Discovery Studio. All ligand partial charges were generated using gaussian03,^a^ and ligand atoms were parameterized according to the GAFF force field (28).

Receptor atomic parameters and partial charges were assigned according to the Amber ff99SB force field (29) using the Amber 10 *xleap* module.^b^ Sodium ions were subsequently added to bring the system to electric neutrality. The protein was then solvated in a TIP3P (30) water box that extended 10 Å beyond the protein in each direction, and additional sodium and chloride ions were added to bring the total salt concentration to 20 mm.

### Molecular dynamics simulations

NAMD 2.6 (31) was used for all minimizations, equilibrations, and free-dynamics runs. Minimization and equilibration steps were performed as described previously by Wang *et al.* (28). In brief, each system was minimized in four phases totaling 45 000 minimization steps. Hydrogen atoms were relaxed in the first 5000 steps; hydrogen atoms and water molecules were relaxed in the next 5000 minimization steps; hydrogen atoms, water molecules, and the atoms of the protein backbone were relaxed in the next 10 000 minimization steps; and all atoms were relaxed for the last 25 000 minimization steps.

For equilibration, 1-ns simulations were performed at 310K using the final minimized structures as the initial coordinates. Harmonic constraints were placed on the atoms of the protein backbones and relaxed in a series of four 250-ps steps. The harmonic restraining force was weakened from 4.0 kcal/mol/Å^2^ in the first 250-ps segment to 3.0, 2.0, and 1.0 kcal/mol/Å^2^ in the following steps, respectively. Before beginning the productive dynamics simulations, each system was checked to ensure that the root-mean-square deviation (RMSD) between the equilibrated and preminimization structures was <1 Å.

For each of the four systems, a 59-ns MD simulation was then performed with a 2-fs time step. Bonds with hydrogen atoms were constrained using the RATTLE algorithm (32), and water geometries were maintained using SETTLE (33), with a bond length error of 0.0005 Å. The temperature bath was kept at 310K with Langevin dynamics. The pressure was maintained at 1 atm using the Nose–Hoover–Langevin piston method (34) with period and decay times set at 100 and 50 fseconds, respectively. Long-range electrostatics were calculated using Particle mesh Ewald (35). The free-dynamics runs were performed on the TACC Ranger supercomputer. A typical benchmark on the 102 911 and 102 884 atom systems was 4.35 nseconds/day of simulation on 96 processors. The system was sampled every 1 pseconds, generating a total of 59 000 coordinate snapshots. For analysis, every 5th frame was used. Each frame was aligned to the first frame of the trajectory by minimizing the alpha carbon root-mean-square (RMS) deviation using the RMSD Trajectory Tool in VMD (25).

### Trajectory clustering

The monomers of each homodimer simulation were isolated, and the two resulting trajectories were concatenated to form a single monomeric trajectory. These monomeric trajectories were subsequently clustered using the gromos algorithm as implemented in the gromos++ analysis software (36). The alpha carbon atoms in the active site, defined as all alpha carbon atoms belonging to a residue that was within 5 Å of the NAD or UDP-sugar in the first frame of the trajectory, were used for the mass-weighted RMSD clustering. The RMSD cutoff was increased by 0.05 Å until the trajectory clustering yielded fewer than 30 clusters, with over 90% of all frames contained in the seven largest clusters.

### Hydrogen bond analysis

Frames extracted from the simulation every 50 pseconds were used for hydrogen bond analysis. The program HBonanza (37) was set to identify all persistent hydrogen bonds present in at least 75% of the frames. Angle and distance cutoffs of 30° and 3.5 Å were used, respectively.

### Principal component analysis

Principal component analysis (PCA) was performed using the *ptraj* module in Amber 10^b^. Normalizing each eigenvalue of the covariance matrix to its total sum yields the percent of all *Tb*GalE movements attributable to the corresponding eigenvector. That is, the largest eigenvalues correspond to the PC modes that best explain the molecular motions sampled by the system trajectories. The principal component projections were visualized using a modified version of the Interactive Essential Dynamics module in VMD (38).

### Identifying highly conserved active site residues

UniProt (39) was used to identify 7142 members of the sugar epimerase family. Only reviewed sequences were considered for subsequent analysis. These sequences were loaded into the MultiSeq (40) module of the VMD molecular graphics computer program (25). MultiSeq was used to generate a non-redundant set of four representative sequences from this input. ClustalW (41) was then used to align these four sequences to the sequence of *Tb*GalE obtained from the PDB structure 2CNB (20). A set of active site residues was obtained by identifying all receptor residues from the 2CNB structure (chain A) that came within 3.5 Å of the cocrystallized NAD^+^ cofactor and the UDP-sugar substrate.

### HsGalE inhibition and thermal scanning fluorimetry assay

Recombinant wild-type *Hs*GalE and human UDP-glucose dehydrogenase (*Hs*UGDH) proteins were expressed in *E. coli* and purified as described previously (42,43). DTP compounds were obtained from the NCI/DTP Open Chemical Repository (http://dtp.cancer.gov).

The rate of the *Hs*GalE-catalyzed reaction was determined by coupling it to the oxidation of UDP-glucose by the action of *Hs*UGDH, essentially as previously described (42). Reaction mixtures (20 nm*Hs*GalE, 1.2 μm*Hs*UGDH, 100 μm UDP-Gal, 10 mm NAD^+^ 10 mm HEPES-NaOH, pH 8.8, 1% (v/v) DMSO) were set up in triplicate with and without DTP compounds (100 μm) in a total volume of 150 μL. The rate of NADH formation was measured at 340 nm for 20 min at 37 °C using a Multiskan Spectrum plate-reader spectrophotometer (Thermo Scientific, Hemel Hempstead, UK) and was used to calculate the rate of production of UDP-glucose by *Hs*GalE. Initial rates were calculated from the linear section of each progress curve and the activity expressed as a percentage of the activity in the absence of the compound.

The binding of the DTP compounds was measured using a thermal scanning fluorimetry assay (44). This assay has been used previously to identify small molecular chaperones for the treatment of phenylketonuria (45), to identify stabilizing additives that facilitate crystallization (46), and to measure the binding affinities of carbonic-anhydrase inhibitors (47). Sypro orange (Sigma, Poole, UK), a fluorescent dye, was diluted from a 5000× solution (manufacturer’s concentration definition) into a 50 × solution with 10 mm HEPES, pH 8.8. This stock solution was mixed well prior to each use. Reactions were carried out in a total volume of 20 μL and contained 5 μm*Hs*GalE, 100 μm DTP compound, 10 mm HEPES, pH 8.8, 1% (v/v) DMSO, 5× Sypro orange. Melting curves were determined using a Rotor-Gene Q cycler (Qiagen, Crawley, UK) with the following protocol: high-resolution melt run (460-nm source, 510-nm detector), 25–95 °C ramp with a 1 °C rise for each step, and no gain optimization. The melting temperatures (*T*_m_) were calculated using the inbuilt analysis software.

## Results and Discussion

### Evidence of simulation equilibration

**Figure 2:**
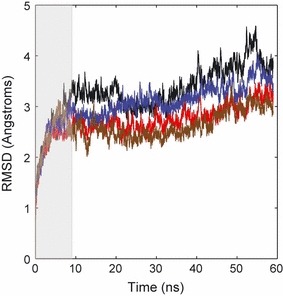
Trajectory RMSD. Each dimer simulation was aligned to the first frame by minimizing the root-mean-square deviation (RMSD) of the Cα’s. RMSD was calculated using the first frame as a reference. For this plot, and in subsequent figures, *apo* is depicted in black, UDP-galactose in blue, UDP-glucose in red, and the UDP-ketose intermediate in brown. As each substrate was derived from UDP-galactose, the systems needed 9 nseconds to equilibrate (gray box). Trajectory analysis was performed on the subsequent 50 nseconds of each dimer simulation.

Alpha carbon RMSD plots of each homodimer trajectory are shown in Figure 2. As the initial protein conformation of each system was that of the crystallographic UDP-galactose-bound state, the first 9 nseconds of each homodimer simulation were discarded to account for system equilibration. The remaining 50 nseconds of the dimeric simulation were used for subsequent analysis. In total, 400 nseconds of productive *Tb*GalE monomer simulation were generated.

### A gating mechanism may mediate ligand binding

**Figure 3:**
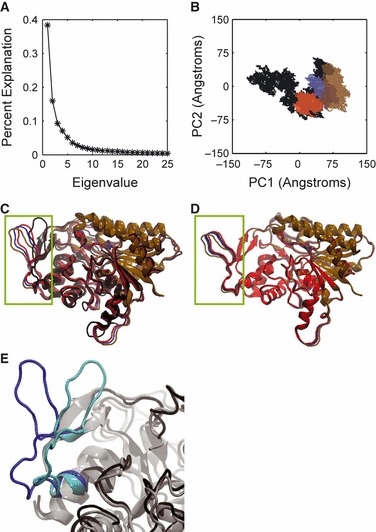
Principal component analysis (PCA). To facilitate comparison, all trajectories were projected onto the principal components of the *apo* simulation. *Apo* is shown in black, UDP-galactose in blue, UDP-glucose in red, and the UDP-ketose intermediate in brown. (A) Percent of the trajectory dynamics attributable to each of the top 25 *apo* eigenvectors. A majority of these motions can be explained by the first two principal components (PC). (B) Projection of the trajectories onto the first two *apo* eigenvectors. Each *holo* simulation explores its own unique set of motions. (C) The PC1 minimum extreme structures for each simulation, color-coded as above. The location of gate is boxed in green. (D) The PC1 maximum extreme structures, color-coded as above. The location of gate is boxed in green. (E) The closed conformation evident in the PC1 minimum *apo* structure results from a kink in the highlighted α-helix. The PC1 minimum structure is shown in cyan/gray, and the maximum is shown in blue/black for reference.

To better understand how receptor dynamics might impact ligand binding, PCA was used to identify the most important molecular motions of each of the four simulations. Principal components, or eigenvectors, were first calculated for the backbone atoms of each trajectory, and each trajectory was subsequently projected onto the *apo* eigenvectors for reference. A majority of the variance in the molecular motions could be explained by the first two principal components, with almost 40% of the variance explained by the first eigenvector alone (Figure 3A). Consequently, only the first two principal components were used for subsequent analysis (Figure 3B).

The Interactive Essential Dynamics computer package (38) was used to identify the extreme structures of each simulation (Figure 3C,D), as judged by projection onto the first *apo* eigenvector. The largest differences in the motions defined by the first principal component were present in the loop region containing residues 230-255. This loop was already thought to be highly dynamic, given that residues 236–248 were unresolved in the 2CNB crystal structure (20).

Much of the difference in the minimum extreme structures can be explained by a kink that forms at the C-terminal end of the α-helix preceding the loop containing residues 230–255 in the *apo* simulation (Figure 3E). This helical bend causes the flexible loop to move toward the active site, as evidenced by the change in the angle defined by the alpha carbon atoms of D245, G228, and R109, which ranges from 67.69° to 36.01° as PC1 decreases, compacting the protein. Additionally, the corresponding distance between D245 and R109 similarly changes from 29.81 to 16.55 Å. The loop may therefore serve as a gating mechanism likely relevant to ligand binding (Figure 3). As the gating loop containing residues 230–255 is near the active site, it is possible that the movement of this loop helps control the UDP-sugar rate of entry.

### UDP-sugar binding: induced fit vs. population shift

The principal components suggest that both induced fit and conformational shift play a role in the binding of *Tb*GalE to its natural substrates. The projection of the *apo* simulation onto the first two principal components demonstrates that the *apo* protein explores a large region of conformational space (Figure 3B). Similar projections of the remaining simulations onto the same *apo* principal components reveal that the UDP-glucose and UDP-galactose simulations sample distinct regions within this larger, *apo* conformational space, suggesting a population shift mechanism of binding. On the other hand, the UDP-ketose intermediate samples a region of conformational space largely unexplored by the *apo* protein; the intermediate itself may therefore provoke receptor conformational changes via an induced-fit mechanism. Limited conformational overlap was expected as the ligands only differ at the UDP-sugar C4 atom; these overlaps likely represent the transitions between states during the actual reaction.

These results suggest a general binding mechanism that may be relevant to the study of other receptor–ligand systems as well. The *apo* protein likely samples a large region of conformational space, occasionally assuming conformations in which the gating loop is open, permitting access to the main, UDP-sugar binding site. When UDP-glucose binds, it stabilizes certain conformations, causing the region of the sampled conformational space to constrict via a population shift mechanism. Next, the bound ligand induces changes in the receptor conformation uncharacteristic of the *apo* protein via an induced-fit mechanism. These conformational changes are likely caused by the transformation of the sugar and are required to accommodate the UDP-ketose intermediate. As the epimerization progresses, the conformational space sampled by the protein returns to that sampled in the *apo* state, again indicative of a population shift mechanism of binding.

Remarkably, the regions of the *apo* conformational space sampled by the UDP-galactose- and UDP-glucose-bound proteins are distinct, despite the fact that these two ligands differ by only a chiral inversion at a single sugar carbon atom, demonstrating that even small differences in bound ligands can drastically change the region of conformational space sampled. This finding has significant implications for computer-aided drug design, as it suggests that the crystallographic and MD-derived structures used in virtual screening can vary remarkably depending on the bound ligand. As previous computer-aided drug discovery efforts directed toward *Tb*GalE only used *holo* simulations to generate receptor structures for virtual screening (12), additional screening against *apo* conformations may reveal new, effective *Tb*GalE inhibitors.

### Hydrogen bonding of the UDP-sugar

**Figure 4:**
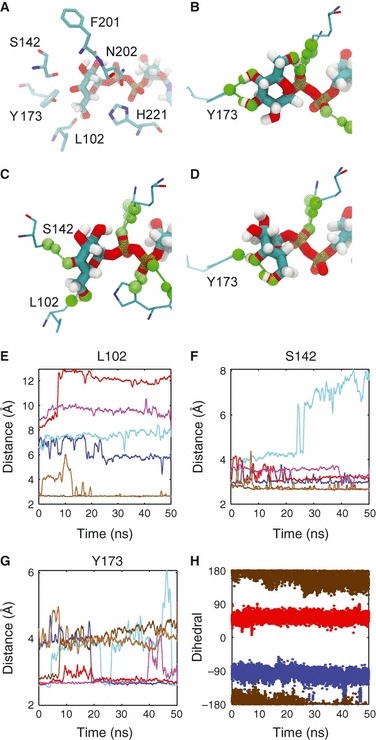
Ligand hydrogen bonding. Hydrogen bonds present in over 75% of the 1000 equidistant frames extracted from each MD simulation are shown in green. In all charts, UDP-galactose is shown in blue, UDP-glucose is shown in red, and the UDP-ketose intermediate is shown in brown. For D–F, chain A is represented by the darker color, and chain B by the lighter color. (A) UDP-glucose. The glucose is held in place by two hydrogen bond interactions with Y173. (B) UDP-ketose intermediate. The orientation of the sugar is flipped from that of UDP-glucose. The sugar C6 hydroxyl group interacts with L102, and the intermediate carbonyl oxygen is tethered to S142. (C) UDP-galactose. The now epimerized sugar, still flipped, forms a single hydrogen bond with Y173. (D–F) Moving average using 500 pseconds windows. (D) Distance from the L102 carbonyl oxygen to the C6 hydroxyl on the UDP-sugar. (E) Distance from the S142 side chain hydroxyl to the C4 hydroxyl on the UDP-sugar. (F) Distance from the Y173 side chain hydroxyl to the C4 hydroxyl on the UDP-sugar. (G) [**C1-O-P-O**] torsion angle of the UDP-sugar. C1 belongs to the sugar.

To examine how the hydrogen bond network of the ligand changes over the course of the reaction, we first identified persistent hydrogen bonds present in over 75% of the trajectory frames analyzed. As expected, the hydrogen bond network that mediates the binding of the UDP-ketose intermediate differs from the networks associated with UDP-galactose and UDP-glucose (Figure 4A–C). Two of the identified hydrogen bonds mediate interactions with Y173 and S142, conserved residues of the catalytic triad that are critical for the two-step GalE epimerization reaction (Figure 1) (11,48).

The hydrogen bond with S142 is particularly interesting, as earlier studies of *Ec*GalE suggested that S142 shuttles a proton to Y173 (49). However, crystallographic studies of several GalE proteins have called into question this theory, as they reveal that the distance between the S142 and Y173 hydroxyl groups is generally large. Our results suggest that the specific role of S142 is to stabilize the UDP-ketose intermediate via hydrogen bonding with the C4 hydroxyl group during the conformational flip required for epimerization (Figure 4B).

The UDP-sugar hydrogen bond networks were generally similar regardless of which monomer was used for analysis, with the exception of UDP-galactose. When acceptor–donor heavy-atom distances were calculated over the course of the trajectories (Figure 4D–F), it was noted that the chain B active site of UDP-galactose underwent a conformational shift approximately 5 nseconds into its trajectory, causing a break in the hydrogen bonds between the UDP-galactose and S142 and Y173, respectively. However, this shift was not observed in chain A, which had a UDP-sugar hydrogen bond network more consistent with the *Tb*GalE crystal structure, 2CNB. Additionally, despite the conformational shift evident in chain B, the key sugar contacts with S142 and Y173 continued to transiently reform during the course of the simulation, confirming that these residues play an important role in stabilizing the UDP-sugar. Other residues, such as N202 and H221, also formed hydrogen bonds with the UDP-sugar, but more transiently (data not shown).

Like S142 and Y173, L102 may also be important for catalysis, warranting further pharmacological study. Our simulations suggest that L102 may be fundamental to the conformational flip required for epimerization (Figure 1). The carbonyl oxygen atom of L102 is predicted to form a persistent hydrogen bond with the C6 hydroxyl group of the UDP-ketose intermediate. Previous crystallographic studies of a UDP-galactose-like ligand suggested that L102 might mediate ligand binding through interactions with the UDP-sugar C6 hydroxyl group (20). Our simulations suggest that an additional function of the L102 backbone carbonyl group is to maintain the UDP-ketose-intermediate in a conformation approximately halfway between that of UDP-galactose and UDP-glucose via a persistent hydrogen bond. The average UDP-sugar [C1-O-P-O] dihedral angle of the UDP-ketose intermediate is approximately halfway between that of UDP-galactose and UDP-glucose (Figure 4G), supporting this notion.

The same hydrogen bond networks that mediate UDP-sugar binding may be germane to the design and optimization of potential small-molecule therapeutics. For example, a common practice in designing inhibitors is to create transition-state analogs. Our simulation of the UDP-ketose intermediate bound to *Tb*GalE has elucidated the important role L102 may play in stabilizing this intermediate. These results suggest that L102 may be a good residue to target in future drug discovery efforts, in addition to the already identified Y173 and S142 (20).

### Identification of conserved residues

As current trypanocidal compounds are subject to growing resistance (50–54), future drug design strategies should also attempt to anticipate mutations that may reduce therapeutic efficacy. One strategy to avoid resistance is to pharmacologically target protein residues that are conserved across related members of the same protein family. Conserved residues are likely to be essential for catalysis and/or the binding of natural substrates and thus are less likely to undergo resistance-conferring mutations.

After considering the 392 reviewed members of the sugar epimerase family listed in UniProt, five representative proteins spanning both the eukaryotic and bacterial domains of life were selected: *Trypanosoma brucei* GalE (PDB ID: 2CNB), a chloroplastic protein from *Arabidopsis thaliana* (UniParc: Q8H124), Gal10 from *Schizosaccharomyces pombe* (UniParc: Q9HDU3), bifunctional polymyxin resistance protein ArnA from *Yersinia enterocolitica* (UniParc: A1JPN5), and probable rhamnose biosynthetic enzyme 1 from *Arabidopsis thaliana* (UniParc: Q9SYM5). The sequences of these five proteins were aligned, and the following *Tb*GalE active site residues were found to be highly conserved: G7, G10, I12, D75, A100, N117, S141, S142, A143, Y173, K177, and R268. Of these, S142, A143, Y173, K177, and R268 appear to associate with the UDP-sugar.

A143 is particularly noteworthy. While A143 did not form a persistent hydrogen bond with the UDP-sugar C3 hydroxyl group in our simulations (i.e., the bond was present in fewer than 75% of all simulation frames analyzed), this hydrogen bond did form transiently, consistent with the 2CNB crystal structure. As S142 and A143 form hydrogen bonds with adjacent UDP-sugar hydroxyl groups, concurrent binding likely serves to stabilize the conformation of the UDP-sugar in a conformation amenable to catalysis. Thus, small-molecule inhibitors that have adjacent hydroxyl groups or similar hydrogen bonding moieties might be good candidates targeting these two conserved residues.

### Allosteric binding

In a recent study, Durrant *et al.* (12) used virtual screening to identify *Tb*GalE ligands from among the compounds of the NCI Diversity Set II. Fourteen low-micromolar inhibitors were ultimately reported. However, in addition to these antagonists, seven agonists, unreported at the time, were also identified (Table S1). We found this result interesting, as the agonists were identified by docking small-molecule models into the *Tb*GalE active site and therefore should compete with the UDP-sugar substrate rather than enhance its catalysis.

**Figure 5:**
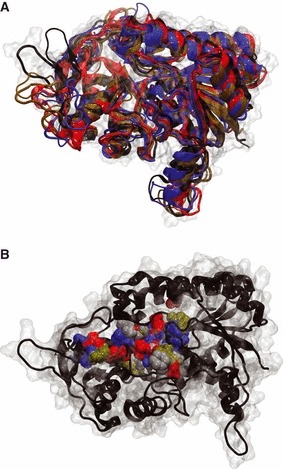
Binding pocket identification. (A) Active site clustering of the *Tb*GalE monomers. The frames of each simulation were clustered by the active site Cα’s using 0.70 and 0.75 Å RMSD cutoffs for the *holo* and *apo* simulations, respectively. The top two representative structures of chain A from each simulation are shown; *apo* is depicted in black, UDP-galactose in blue, UDP-glucose in red, and the UDP-ketose intermediate in brown. Darker and lighter colors correspond to the most populated and the second most populated cluster, respectively. (B) FT-MAP analysis. The top five clusters from each chain were submitted to the FT-MAP server. Shown are the results for the top chain A cluster of each simulation. These results suggest that *Tb*GalE contains no allosteric sites; the observed agonism likely results from ligand-induced dimerization and/or cooperativity between the two monomers.

Although *Tb*GalE agonists are ill suited as HAT therapeutics, a human condition known as type III galactosemia arises from a deficiency in *Hs*GalE. Understanding GalE agonism is therefore of great therapeutic interest. To determine whether agonism might arise from allosteric binding, the *holo* and *apo* monomer trajectories were clustered into five groups using RMSD cutoffs of 0.7 and 0.75 Å, respectively (Figure 5A). A representative protein conformation was then taken from each cluster; together, these representative conformations are said to constitute an *ensemble*.

The FT-MAP server (55) was then used to computationally flood the entire surface of the ensemble conformations with models of small organic probes in an attempt to identify potential binding pockets. FT-MAP identified the NAD(H) and UDP-sugar binding pockets in every structure; however, no other potential allosteric pockets were consistently recognized (Figure 5B), suggesting that agonism does not likely occur by allosteric modulation.

While the possibility of allosteric binding at an uncharacterized secondary site could not be ruled out, the fact that FT-MAP revealed no such site suggests cooperative agonism mediated by binding to the principal site. It may be that agonist binding to one *Tb*GalE monomer alters the affinity of the binding site on the other dimeric monomer, thus facilitating additional binding. The steep Hill coefficients associated with several of the inhibitors, even in the presence of a detergent that disrupts colloidal aggregates, support this theory (56,57). Hemoglobin is the classic example of cooperative binding, but many other examples exist in nature as well. As an alternate explanation, agonist binding to the principal site of one *Tb*GalE monomer might drive dimerization by stabilizing the four-helix bundle at the dimer interface. This theory is supported by previous evidence in related proteins suggesting that, while the monomer is functional, full functionality is achieved only through dimerization (58,59).

Unfortunately, when the identified *Tb*GalE agonists were tested against *Hs*GalE, no agonism was noted (Figure S1, Tables S2 and S3). However, it is possible that the lack of activity arose from differences in the human and *T. brucei* versions of the GalE protein rather than fundamental differences in the agonistic mechanism. For example, the NAD^+^ adenine-binding pocket is more closed in *Tb*GalE, and *Hs*GalE G237 in the UDP-sugar binding domain is replaced by C266 in *Tb*GalE, a potentially reactive residue that may prove useful in future drug design efforts (*11*). Further efforts to identify *Hs*GalE agonists may therefore be justified.

## Conclusions and Future Directions

In this work, we have used molecular simulations to probe the dynamics of *Tb*GalE and to specifically analyze the mechanisms governing ligand-binding and enzymatic conversion. Our MD simulations suggest that the conformations sampled by *Tb*GalE are highly dependent on the composition of the ligand, as even the chirality of the UDP-sugar C4 atom greatly affected the conformations explored.

Additionally, we have identified an active site residue, L102, that may be important in the stabilization of the UDP-ketose intermediate. While this residue has been previously identified in crystallographic studies as a potential mediator of UDP-galactose binding (20), to our knowledge, its possible role in stabilizing the UDP-ketose intermediate has not been previously recognized.

Finally, as none of our simulations revealed any *Tb*GalE allosteric sites, we anticipate that dimeric agonism likely results from either cooperative binding or dimeric stabilization. We hoped that the results presented here will not only provide insight into the function of this and related enzymes, but also assist future computer-aided drug discovery efforts targeting *Tb*GalE and *Hs*GalE.

## Footnotes

a Frisch MJ, Trucks GW, Schlegel HB, Scuseria GE, Robb MA, Cheeseman JR, *et al.* Gaussian 03, Revision C.02.

b Case DA DT, Cheatham TE III, Simmerling CL, Wang J, *et al.* (2008) AMBER 10. San Francisco: University of California.

## Abbreviations

*Tb*GalE: *Trypanosoma brucei* UDP-galactose-4′-epimerase
HAT: Human African trypanosomiasis
MD: molecular dynamics
RMSD: root-mean-square deviation
PCA: principal component analysis
*Hs*UGDH: human UDP-glucose dehydrogenase

## Supporting Information

**Figure S1.** Thermal scanning fluorimetry of*Hs*GalE.

**Table S1.** *Tb*GalE Agonists

**Table S2.** Percentage activity of 20 nM *Hs*GalE in the presence of different DTP compounds

**Table S3.** Melting temperatures of *Hs*GalE in the presence of different DTP compounds
