# Supplementary material for: The Molecular Dynamics of Trypanosoma brucei UDP-Galactose 4′-Epimerase: A Drug Target for African Sleeping Sickness
Source: Chem Biol Drug Des. 2012 Aug;80(2):173–81. doi: 10.1111/j.1747-0285.2012.01392.x (PMC3399956; doi:10.1111/j.1747-0285.2012.01392.x)
Supplement: Supplementary file 1 [file cbdd0080-0173-SD1.doc]

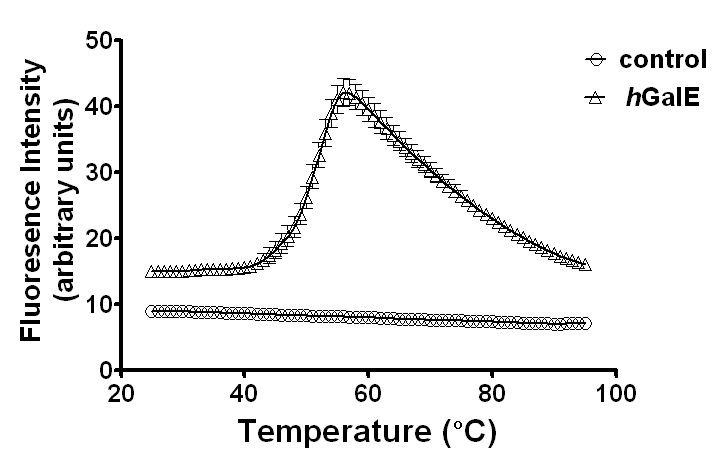


**Figure S1. Thermal scanning fluorimetry of *Hs*GalE.** 5 μM *Hs*GalE in 10 mM HEPES-NaOH, pH 8.8, 1% (v/v) DMSO, 5× Sypro orange showed a clear melting curve resulting in a T_m_ of 51.5±0.3 °C.

**Table S1. *Tb*GalE Agonists**

| **NSC ID** | **Structure** | **% inhib. @ 100 mM** |
| --- | --- | --- |
| **91395** | 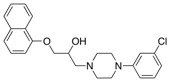 | **-167** |
| **61610** | 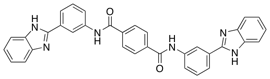 | **-169** |
| **7524** | 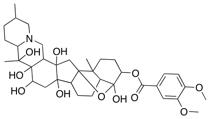 | **-191** |
| **91396** | 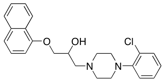 | **-194** |
| **260594** | 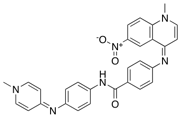 | **-223** |
| **146771** | 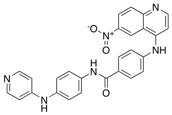 | **-242** |
| **202386** | 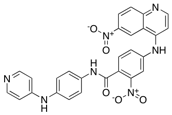 | **-283** |

Specific methods can be found in Durrant et al. (2010) *J Med Chem 53*, 5025-5032.

**Table S2. Percentage activity of 20 nM *Hs*GalE in the presence of different DTP compounds**

| **DTP Compound** | **% Activity** |
| --- | --- |
| No compound | 100 ± 17 |
| 91395 | 102 ± 32 |
| 61610 | 89 ± 27 |
| 7524 | 112 ± 3 |
| 91396 | 124 ± 30 |
| 260594^a^ | 30 ± 190 |
| 146771^a^ | 104 ± 57 |
| 202386^a^ | 41 ± 59 |

The reactions contained 100 μM DTP compound, 100 μM UDP-Galactose, 10 mM NAD^+^, 1.2 μM HsUGDH, 10 mM HEPES-NaOH, pH 8.8, 1% (v/v) DMSO. Data are reported as the mean ± SD determined from three separate experiments. No compound resulted in a statistically significant (Student’s t-test) change in activity.

^a^Compounds 260594, 146771 and 202386 gave large errors due the formation of a coloured precipitate, which prevented accurate determination of activity.

**Table S3. Melting temperatures of *Hs***GalE in the presence of different DTP compounds

| **DTP Compound** | **T_m_ (°C)** | **ΔT_m_ (K)** |
| --- | --- | --- |
| No compound | 51.5 ± 0.3 | N/A |
| 91395 | 51.3 ± 0.3 | − 0.2 ± 0.6 |
| 61610 | 51.4 ± 0.1 | − 0.1 ± 0.4 |
| 7524 | 51.3 ± 0.4 | − 0.2 ± 0.7 |
| 91396 | 51.3 ± 0.3 | − 0.2 ± 0.6 |
| 260594^a^ | N/D | N/D |
| 146771^a^ | N/D | N/D |
| 202386^a^ | N/D | N/D |

The reactions contained 5 μM *Hs*GalE, 100 μM DTP compound, 10 mM HEPES, pH 8.8, 1% (v/v) DMSO, 5× Sypro orange. The change of melting temperature, ∆T_m_, due to ligand binding was calculated according to:

∆T_m_ **=** (T_m_ of protein without compound) - (T_m_ of protein with compound)

Data are reported as mean ± SD determined from three experiments. If a compound bound to the enzyme, it would be expected to stabilize the protein’s structure resulting in an increase in T_m_. However, none of the compounds tested here resulted in a statistically significant (Student’s t-test) change in T_m_.

^a^ Compounds 260594, 146771 and 202386 formed a colored precipitate, preventing determination of the melting temperature.
